# Neuropeptide Y directly reduced apoptosis of granulosa cells, and the expression of NPY and its receptors in PCOS subjects

**DOI:** 10.1186/s13048-023-01261-8

**Published:** 2023-08-31

**Authors:** Yoko Urata, Reza Salehi, Brandon A. Wyse, Sahar Jahangiri, Clifford L. Librach, Chii-Ruey Tzeng, Yutaka Osuga, Benjamin Tsang

**Affiliations:** 1 Departments of Obstetrics & Gynecology and Cellular & Molecular Medicine, Interdisciplinary School of Health Sciences, University of Ottawa, Ottawa, Canada; 2 Chronic Disease Program, Ottawa Hospital Research Institute, Critical Care Wing, 3rd floor, Room W3107, 501 Smyth Road, Ottawa, ON K1H 8L6 Canada; 3 Department of Obstetrics and Gynecology, the University of Tokyo, 7-3-1 Hongo, Bunkyo-Ku, Tokyo, 113-8655 Japan; 4 CReATe Fertility Centre, Toronto, ON Canada; 5 Departments of Obstetrics & Gynaecology and Physiology, Institute of Medical Sciences, University of Toronto, Toronto, ON Canada; 6 Biological Sciences, DAN Women & Babies Research Program, Sunnybrook Research Institute, Toronto, Canada; 7 Center for Reproductive Medicine and Science, Taipei Medical University Hospital, Taipei, Taiwan

**Keywords:** Neuropeptide Y, Neuropeptide, Apoptosis, Hyperandrogenism, Polycystic ovarian syndrome

## Abstract

**Background:**

Most women with anovulatory infertility show polycystic ovarian syndrome (PCOS), and androgen excess is known as a key factor involved in pathogenicity of PCOS. However, the mechanism of follicular developmental arrest in PCOS is not completely understood. The reproductive function of Neuropeptide Y (NPY) in the ovary during folliculogenesis was previously reported; NPY function in apoptosis and proliferation of granulosa cells (GCs) is follicular-stage dependent. The objective of this study was to investigate the role of NPY in ovarian follicular development and the pathogenesis of PCOS.

**Methods:**

To simulate the PCOS phenotype using a rat model, 21-day old Sprague Dawley rats were implanted with dihydrotestosterone (DHT) capsule (83 µg/day) and euthanized after 28 days. mRNA and protein content of NPY and its receptors were assessed in GCs from DHT treated rats using RT-qPCR and Western blot, respectively. Proliferation and apoptosis of GCs was assessed using Ki67- and TUNEL assays. Finally, NPY levels were measured in human follicular fluid (FF) from matched PCOS and non-PCOS patients using ELISA.

**Results:**

GCs from DHT treated rats (PCOS-GCs) contained significantly less NPY protein and *Npy* mRNA by 0.16- and 0.56-fold, respectively, and more NPY receptor type 2 and 5 protein by 2.21- and 3.17-fold, respectively, when compared to sham control. Addition of recombinant NPY to PCOS-GCs culture did not alter Ki67-positive but significantly decreased TUNEL-positive cells by 0.65-fold, but not to baseline levels. There was no significant difference in NPY levels in FF between PCOS and non-PCOS subjects.

**Conclusions:**

These results indicate that DHT modulates expression of NPY and its receptors, NPY decreases DHT-induced GCs apoptosis. That alterations in NPY’s function might be involved in follicular developmental failure of PCOS.

**Supplementary Information:**

The online version contains supplementary material available at 10.1186/s13048-023-01261-8.

## Background

Polycystic ovarian syndrome (PCOS) is the most common hormonal disorder in women and affects 6 to 10% of women of reproductive age [1]. PCOS accounts for ~ 80% of women with anovulatory infertility [2], and is characterized by anovulatory infertility, hyperandrogenemia, obesity and impaired glucose tolerance, and associated with cardiovascular abnormality and endometrial hyperplasia. PCOS is heterogeneous and, according to Rotterdam criteria, is confirmed if two of the three features—hyperandrogenism, ovulatory dysfunction and polycystic ovarian morphology—are evident [3]. The characteristic morphological feature of polycystic ovaries is accumulation of antral follicles (5–8 mm) and cessation of follicular growth [4, 5].

Androgen excess is a key factor in the pathogenesis of PCOS [6], and this notion has been supported by observations from a number of animal models exhibited hyperandrogenemia [7–10]. For example, dihydrotestosterone (DHT)-treated rats exhibit of metabolic phenotypes such as increased body weight, body fat, insulin resistance and dysregulated cardiovascular control [7, 11, 12]. In addition, human and animal studies indicate that the etiology of PCOS involves the participation of not only genetic, epigenetic, metabolic and inflammatory processes but also neuroendocrine factors [13], although its pathogenesis remains unclear.

Neuropeptide Y (NPY) is a 36-amino acid regulatory peptide and five mammalian NPY receptors (Y1, Y2, Y4, Y5 and y6) have been cloned. All of Y1, Y2, Y4 and Y5 receptors are coupled to an inhibitory G protein, except the y6 receptor which is truncated in the most mammals including human, but is functional in mice [14]. NPY is abundant in the neural system [15] and is involved in the regulation of energy homeostasis [16], memory retention [17] and feeding [18] and reproductive behaviors [19]. NPY is known to play a direct regulatory role on ovarian granulosa cells [20] and we previously reported that NPY induced proliferation of granulosa cells of preantral and early antral follicle and apoptosis of those of late antral follicles [21]. A clinical association between serum NPY level with PCOS has been reported that non-obese women with PCOS had higher plasma level of NPY than those without PCOS [22, 23] while non-obese and obese adolescents with PCOS had higher plasma level of NPY than those without PCOS [24]. The association has only been demonstrated in observational studies using clinical samples, and there are no reports from basic science on a possible role of ovarian NPY in the pathogenesis of PCOS.

The objective of this study was to investigate whether (a) hyperandrogenism is associated with altered expression of NPY and its receptors in granulosa cells; (b) these changes influence granulosa cell fate (proliferation versus apoptosis); and (c) the follicular fluid content of NPY is associated with PCOS. We hypothesized that androgen excess alters the expression of NPY and its receptors in granulosa cells and that their expression pattern determine whether NPY will promote granulosa proliferation or apoptosis in autocrine manner. We also propose that the pathogenesis of PCOS is associated with altered NPY level in follicular fluid. To test this hypothesis, we analyzed the protein levels of NPY and its receptors in granulosa cells isolated from DHT-treated rats in vivo. We also investigated the effect of NPY on granulosa cell proliferation and apoptosis in vitro and compared NPY level in human follicular fluid from PCOS and non-PCOS subjects.

## Results

### NPY protein and Npy mRNA in granulosa cells decreased in DHT-treated rats

**Fig. 1:**
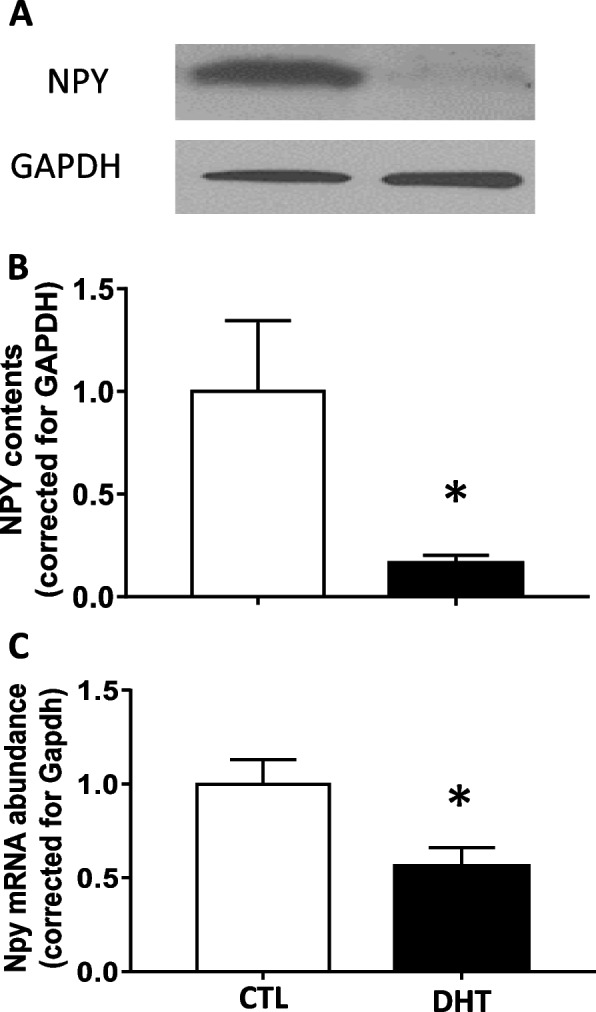
DHT in vivo treatment reduced NPY contents and Npy mRNA abundance in isolated granulosa cells. Granulosa cells of in vivo DHT treatment model rats were isolated. **A**, **B** Total protein was extracted and NPY contents were assessed by Western blot. Representative Western blot result is shown (**A**). **C** Total mRNA was extracted, reverse transcribed and amplified by real-time PCR using specific primers for *Npy* and *Gapdh*. Expression of *Npy* mRNA was normalized to RNA loading for each sample using *Gapdh* mRNA as an internal standard. DHT treatment significantly reduced *Npy* mRNA abundance and NPY content granulosa cells. These results indicate that hyperandrogenism reduces NPY expression in granulosa cells. One replicate of granulosa cells was isolated from one rat. Results are expressed as mean ± SEM of independent replicates (**A**, **B**; CTL, *n* = 4; DHT, *n* = 4) (**C**; CTL, *n* = 6; DHT, *n* = 4) and analyzed by Welch’s test (**B**) and unpaired *t* test (**C**). *, *p* < 0.05

To determine whether androgen regulates granulosa cell NPY expression in vivo, granulosa cells were isolated from both DHT-treated and control rats, and NPY protein content and mRNA abundance were assessed by Western blot and real time PCR, respectively. Both of NPY protein (Fig. 1A and B) and mRNA (Fig. 1C) were significantly reduced by 0.16- and 0.56-fold, respectively in the DHT treated group compared to the control.

### NPY receptor content in granulosa cells were upregulated in a subtype-specific manner in DHT-treated rats

**Fig. 2:**
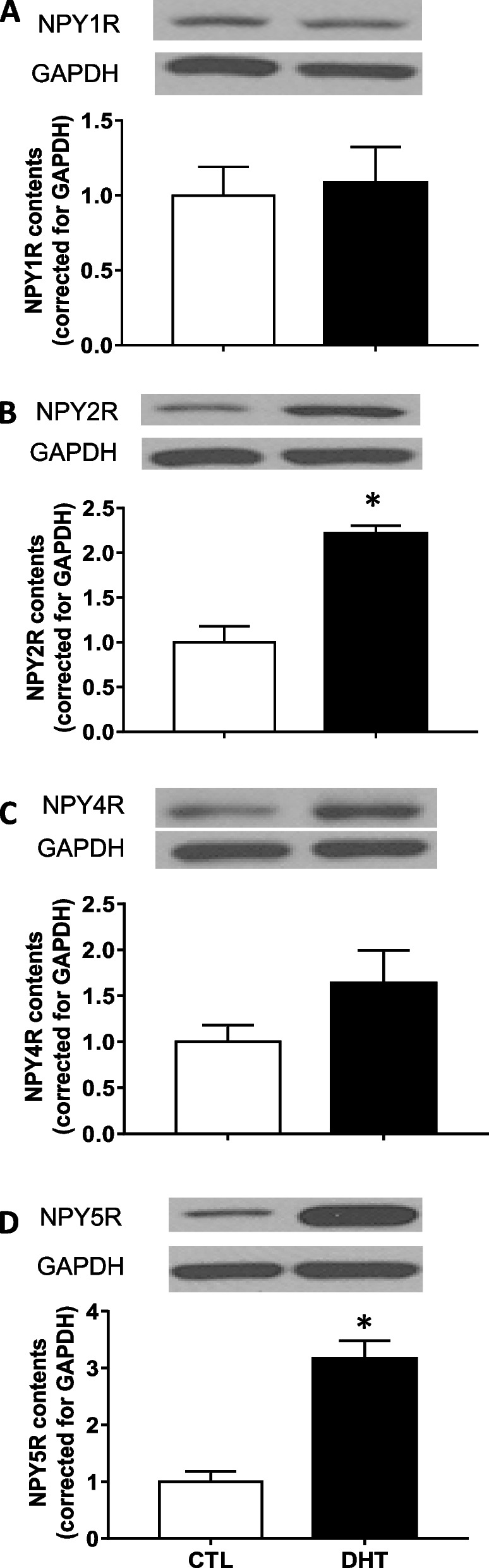
DHT in vivo treatment regulated NPY receptors contents in granulosa cells. Granulosa cells of in vivo DHT treatment model rats were isolated. Total protein was extracted and NPY receptors contents were assessed by Western blot. Representative Western blot results are shown. DHT treatment significantly increased NPY2R (B) and NPY5R (D) contents in granulosa cells. These results indicate that hyperandrogenism regulate NPY receptors in a receptor-type-specific manner. One replicate of granulosa cells was isolated from one rat. Results are expressed as mean ± SEM of independent replicates (CTL, *n* = 6; DHT, *n* = 6) and analyzed by unpaired *t* test. *, *p* < 0.05

To determine whether androgen alters granulosa cell NPY receptor content, granulosa cells from DHT-treated rats were assessed for NPY receptor protein content. NPY receptor Y2 (NPY2R) and Y5 (NPY5R) protein content in granulosa cells from DHT-treated rats were increased by 2.21- and 3.17-fold compared to those in sham control rats, respectively (Fig. 2B and D; *p* < 0.05). In contrast, NPY receptor Y1 (NPY1R) and Y4 (NPY4R) proteins in granulosa cells were not changed in DHT-treated rats (Fig. 2A and C) suggesting that androgen excess increases NPY receptor expression in a subtype-specific manner.

### NPY reduces apoptosis in granulosa cells from DHT-treated but not from control rats

**Fig. 3:**
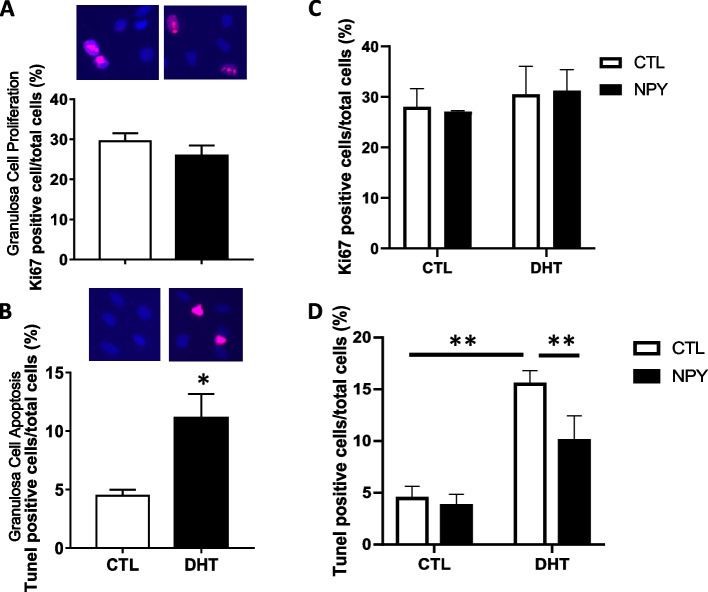
NPY reduced TUNEL-positivity of granulosa cells of DHT-treated rats, whereas NPY did not affect proliferation of granulosa cells. Granulosa cells were isolated from in vivo DHT treatment model rats, incubated with 10% serum overnight and analyzed Ki67 positivity for proliferation (A) and TUNEL assay for apoptosis (B). Representative pictures are shown **A**, **B** Isolated granulosa cells from in vivo DHT treatment model rats were starved for 24 h and treated with NPY (0.1 nM) in vitro for 24 h. Their Ki67 and TUNEL positivity were analyzed **C**, **D** DHT and NPY treatment did not affect Ki67 positivity. NPY in vitro treatment did not change TUNEL-positivity in CTL rats, but reduced that in DHT-treated rats. These results suggested hyperandrogenism change the NPY-mediated apoptosis in granulosa cells. Results are expressed as mean ± SEM of independent replicates (A,B; CTL, *n* = 6; DHT, *n* = 6) (C,D; CTL, *n* = 3; DHT, *n* = 3) and analyzed by unpaired t test (A,B) and two-way ANOVA following multiple comparison, Bonferroni (C,D). *, *p* < 0.05 (vs. with DHT, in vivo). **, *p* < 0.05 (vs. with DHT, in vivo*, without NPY*, in vitro*)*

To investigate whether NPY regulates granulosa cell fate (proliferation vs. apoptosis) and whether this response is influenced by androgen treatment in vivo, rats were treated with DHT and granulosa cells were isolated and incubated with NPY in vitro and granulosa cell proliferation (Ki67) and apoptosis (TUNEL) were assessed. DHT did not significantly affect granulosa cell proliferation in vivo (Fig. 3 A), it increased the apoptotic rate by 2.5-fold compared to sham control in vivo (Fig. 3 B; *p* < 0.05). Next, the influence of NPY on proliferation and apoptosis of granulosa cells from DHT treated rats was evaluated in vitro. The treatment of granulosa cells with NPY did not alter Ki67-positivity irrespective of DHT treatment in vivo (Fig. 3 C; *p* > 0.05). While NPY did not alter TUNEL-positivity of granulosa cells in sham control rats, it significantly reduced the number of TUNEL positive cells in the DHT group by 0.65-fold (Fig. 3D; *p* < 0.05), suggesting that hyperandrogenism alters NPY-mediated apoptosis in granulosa cells.

### Follicular fluid NPY concentration of women without PCOS weakly tended to correlate with BMI

**Fig. 4:**
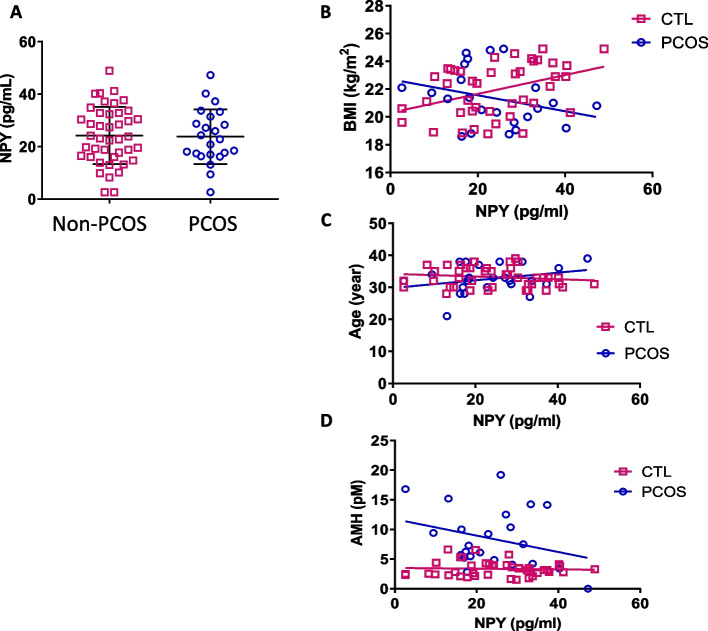
NPY concentration in human follicular fluid of women without PCOS was weakly tended to correlate with BMI. Follicular fluid was collected from single leading follicle at egg-retrieval process and assessed NPY level using a specific ELISA kit. Recruited IVF patients were under 40 years old and had normal body weight (BMI 18.5–24.9). NPY level in follicular fluid of non-PCOS had a significant weak correlation with BMI positively, while those of PCOS did not. Each dot indicates an individual result (non-PCOS, *n* = 42; PCOS, *n* = 23) (**A**, **B**, **C**, **D**), bars indicate mean and SD (**A**) and regression curve (**B**, **C**, **D**). Data were analyzed by unpaired t test (**A**) and Pearson’s test (**B**, **C**, **D**)

To investigate whether NPY in human ovarian follicles is associated with pathogenesis of PCOS, we compared the NPY concentration of follicular fluid and serum of the same patients, and evaluated the NPY concentration in follicular fluids from IVF patients with and without PCOS. We had 33 paired samples of serum and FF from the same patient. The assessment of paired samples showed that NPY concentration follicular fluid was higher than serum (mean ± SD; all women (*n* = 33), 20.1 ± 6.4 vs. 14.0 ± 3.7 pg/ml, *p* < 0.001; non-PCOS subjects (*n* = 23), 19.7 ± 6.3 vs. 14.1 ± 3.6, *p* < 0.001; PCOS subjects (*n* = 10), 20.9 ± 6.8 vs. 14.0 ± 4.3, *p* < 0.01). NPY concentration was not significantly different in follicular fluid between women without or with PCOS (24.2 ± 10.9 vs. 23.8 ± 10.4 pg/ml, mean ± SD; *p* = 0.437; Fig. 4A). We observed a weak significant correlation between NPY FF levels and BMI (*p* = 0.005, *r* = 0.393) in women without PCOS, but not in women with PCOS (*p* = 0.085, *r* = -0.295; Fig. 4B). NPY concentration was not significantly correlated with age (*p* = 0.175, *r* = -0.147; *p* = 0.094, *r* = 0.283; Fig. 4C) nor with serum Anti-Müllerian hormone (AMH) levels (*p* = 0.371, *r* = -0.0535; *p* = 0.088, r = -0.291; Fig. 4D) among women without or with PCOS.

## Discussion

Pathogenesis of follicular developmental failure in PCOS is not fully understood. In the present study, we have demonstrated that (a) in DHT-treated rats the expression of NPY in granulosa cells was reduced while its receptors NPY2R and NPY5R were upregulated; (b) NPY treatment reduced apoptosis in granulosa cells of DHT-treated but not sham-control rats; and (c) PCOS was not significantly associated with the follicular fluid level of NPY. These results suggest that dysregulated NPY receptor expression may be an important etiological factor in follicular developmental failure in PCOS.

We found a lower NPY expression in granulosa cells isolated from hyperandrogenized PCOS rat model. In several PCOS animal models, chronic androgen excess induces NPY expression in arcuate nucleus of the hypothalamus and *Npy* mRNA abundance in adipose tissue [9, 10, 25]. On the other hand, Jana et al. reported that postnatal treatment of testosterone in gilts reduced both NPY-immunoreactivity and total nerve fibers in ovaries [26]. In human, serum NPY level in normal weight PCOS women was higher than those in non-PCOS women [22, 23]. Guzelkas et al. reported that non-obese and obese adolescents with PCOS had higher plasma level of NPY than those without PCOS [24]. However, there are no reports demonstrating how NPY functions at the ovarian level of PCOS in either animal models or human studies. In the present study, we observed for the first time that postnatal DHT treatment reduced NPY expression in granulosa cells, which is consistent with NPY level in ovarian nerve fiber [26] and in contrast with that in the central neuron system [9, 10]. The tissue-specific variation of NPY expression suggests that NPY may function in both paracrine and autocrine manners. However, paracrine and autocrine actions of NPY may have distinct functions, and it is possible that the mechanism of NPY expression and function may be regulated by a combination of central neuron and peripheral tissues. This notion is in accordance with our previous report showing NPY expression in granulosa cells is follicular stage-dependent [21] with no difference in serum NPY level between luteal and follicular phases [27].

In addition to NPY expression, we have demonstrated that chronic DHT treatment in vivo increased granulosa cell NPY2R and NPY5R expression which may affect NPY-mediated granulosa cell apoptosis. In diabetic patients, upregulation of NPY2R and NPY5R in myocardium were associated with angiogenesis, apoptosis, and vascular smooth muscle proliferation [28]. Acute endometritis induced NPY2R in gilt myometrium, and affected uterine contractility [29]. Glucocorticoid increased NPY2R in adipocytes of the abdominal fat and induced angiogenesis and adipogenesis, resulting in obesity and metabolic syndrome [30]. Further, it has been demonstrated that the interaction of NPY2R/NPY5R regulate cell proliferation [31, 32]. NPY2R is reported to be associated with leptin-induced granulosa cell apoptosis [33]. The elevation of granulosa cell NPY2R and NPY5R expression in DHT-treated rats leads us to speculate that DHT might be a regulator of NPY2R and NPY5R expression. However, the precise mechanism remains to be determined.

In the present study, chronic DHT treatment in vivo also induced apoptosis in granulosa cells, consistent with previous report on acute in vitro DHT treatment [34] and chronic in vivo DHT treatment [35]. NPY did not affect proliferation and apoptosis in granulosa cells of the control sham rat ovary, which differs from our previous report that NPY increased proliferation but not apoptosis of early antral follicle granulosa cells [21]. In this study, ovarian follicular development was not synchronized by eCG, and unsynchronized granulosa cells might have a different expression pattern of NPY receptors from synchronized granulosa cells, which could account for the differences.

We showed that NPY level in human follicular fluid had no significant association with PCOS while NPY level of follicular fluid of PCOS showed different tendency of correlation with BMI, age and serum AMH level. The presence of NPY in follicular fluid has been demonstrated [36], although whether and how it contributes in the pathogenesis of follicular development failure are unknown. Recently, we and Sirotkin et. al. reported that NPY plays an important regulatory role in granulosa cell apoptosis and proliferation [20, 21]. In the ovary, analgesia induced by the combination of electro-acupuncture and paracervical blocks increased NPY level in follicular fluid and improved IVF outcome [37]. However, in present study, no difference was observed in NPY level in human follicular fluid between non-PCOS and PCOS, while NPY contents of granulosa cells from DHT-treated rats was decreased. In granulosa cells of human PCOS, NPY function may be regulated by expression pattern of NPY receptors. This discrepancy is likely due to the fact that the source of NPY in follicular fluids may not be supplied by granulosa cells alone but may include other sources such as cumulus, peripheral blood and nerve may contribute to and wash out the dynamics observed in the in vitro GC culture model presented here. Moreover, human follicular fluid during IVF procedure is collected at the late antral/periovulatory follicles, while chronic in vivo DHT-treated rats granulosa cells are mainly collected from early antral follicles. This difference in follicular stages might also contribute to the latter discrepancy. We have demonstrated that NPY concentration of follicular fluid was higher than the serum using paired samples from the same patient suggesting that NPY is locally produced and may have a follicle-specific function. Using chronically androgenized PCOS rat model, we have demonstrated that NPY suppresses androgen–induced apoptosis of granulosa cells, indicating that NPY in follicular fluids may contribute to pathogenesis of follicular developmental failure in PCOS.

A limitation of this study is that hyperandrogenism can only partially explain the pathophysiology of PCOS. Based on the Rotterdam criteria, women with PCOS are diverse, and those without hyperandrogenism can be diagnosed with PCOS. In the present study we used DHT-treated rats as PCOS model while our human samples may include women without hyperandrogenism. Thus, we cannot attribute the effects observed in humans solely to hyperandrogenism alone. In the future, we plan to further investigate NPY function using a PCOS animal model without androgen administration to better understand the pathological role of NPY in PCOS.

**Fig. 5:**
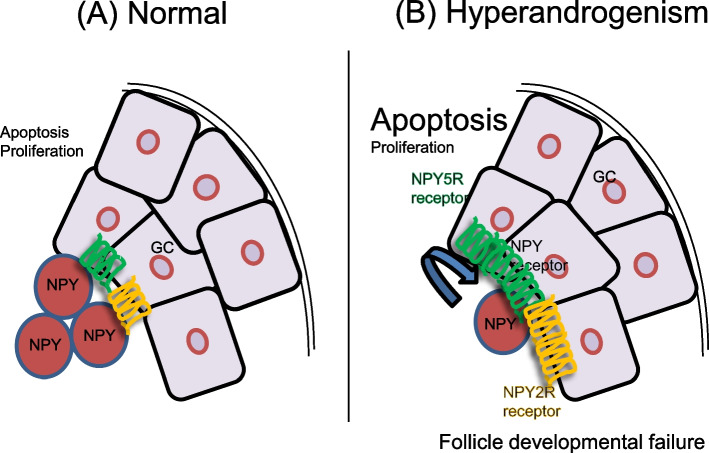
A hypothetical model illustrating the function of hyperandrogenism and NPY regulating granulosa cells. **A** In normal condition, NPY has no significant influence on proliferation nor apoptosis. **B** Hyperandrogenism reduces NPY content in granulosa cells and induces NPY2R and NPY5R contents in granulosa cells. Under hyperandrogenism, NPY does not affect proliferation but reduces apoptosis of granulosa cells in autocrine manner. NPY2R or NPY5R might be involved in NPY-reduced apoptosis. NPY may contribute to follicle developmental failure of PCOS

In conclusion, we have demonstrated for the first time a possible role of NPY in the follicular developmental failure in the pathogenesis of PCOS. Hyperandrogenism altered both expression and function of NPY in granulosa cells. To facilitate the future investigation on a role of NPY in pathogenesis of PCOS, we propose the following working hypothesis: androgen excess, as often observed in PCOS, reduces NPY production and induces NPY2R and NPY5R expression in granulosa cells. In hyperandrogenism, NPY in follicular fluid reduced androgen-induced apoptosis in autocrine manner as NPY receptors expression are higher, but not in the normal condition (Fig. 5). These studies may lead to understand molecular and cellular pathogenesis of follicular development failure in PCOS. As a result, new therapeutic target of follicular developmental failure including PCOS could be developed.

## Material and methods

### Reagents and materials

The reagent and material sand the antibodies used in this study are described at Suppl. Tables S1 and 2, respectively.

### Chronically androgenized rat model

Female Sprague Dawley rats were obtained from Charles River Canada (Montreal, QC, Canada), and all animal procedures were carried out in accordance with the Canadian Council on Animal Care Guidelines and approved by the University of Ottawa Animal Care Committee (Protocol # OHRI-1624). Female 21-day-old rats were implanted subcutaneously with silicone capsules without (sham control) or with DHT continuously released (83 μg/day), as previously described [35, 38]. Rats were sacrificed 28 days after implants.

### Granulosa cell isolation

Ovaries were collected from DHT-treated rats and sham controls and preincubated with 6 mM EGTA and 0.5 M sucrose [39]. Granulosa cells were released by follicular puncture with a 28-gauge needle, washed, and centrifuged (200 × g, 5 min). During ovarian puncture, the follicle morphology was checked under a microscope and only early antral follicles were punctured. Cell clumps and oocytes were removed by filtering the cell suspensions through a 40-μm nylon cell strainer. The number of viable granulosa cells was determined by trypan blue exclusion. Granulosa cells were plated overnight (35,000 per well in a chamber slide) in M199 containing 100 U/mL of penicillin–streptomycin and 1.25  g/mL of amphotericin B with 10% FBS under a humidified atmosphere of 95% air and 5% CO_2_. Following overnight culture, cells were starved for 24-h in serum-free culture medium, then treated with NPY (0.1 nM) for 24-h based on previous study [21]. Following NPY treatment, in a subset of wells cells were lifted for western blotting and qPCR assessment of NPY and NPY receptor expression, described below; the remainder of the wells were assessed for Ki67-positivity and TUNEL assay as described below [40].

### Western blotting

Granulosa cells were lysed in cell lysis buffer containing cOmplete and phosSTOP. Cells were sonicated and lysates were centrifuged (20 min, 15,000 × g, 4 °C). Protein concentration was determined with the Bradford assay (Bio-Rad DC Protein Assay Reagent). To analyze NPY and NPY receptors contents, 30 µg and 20 µg protein lysates were separated by 16.5% Tricine-SDS-PAGE and 10% SDS-PAGE, respectively. NPY protein molecular weight is 11 kDa, requiring greater amount of loading protein to obtain clear signal than NPY receptors. Separated proteins were electrotransferred to PVDF membranes (for NPY) and nitrocellulose membranes (for NPY receptors). Nonspecific binding to the membranes were blocked with skim milk [5%, 1 h, room temperature (RT)]. Blots were incubated with primary antibody (overnight, 4 °C) and then with HRP-conjugated secondary antibody (1 h, RT). Peroxidase activity was visualized with an enhanced chemiluminescence kit, and membranes were exposed to X-ray film. Signals were densitometrically quantified using FIJI software. Antibodies used in this study are described at Suppl. Table 1.

### RNA extraction, reverse transcription, and real-time PCR

RNA was extracted from granulosa cells using RNeasy mini kit. One µg of total RNA was reverse transcribed in 20-µl, using a High-Capacity cDNA Reverse Transcription Kit (ThermoFisher). Real-time PCR was run using Light Cycler ® 480 SYBR Green I (Roche Diagnostics GmbH) (45 cycles at 95 °C (10 s), 60 °C (10 s), 72 °C (7 s)). *Npy* expression was normalized to *Gapdh* and fold change was assessed the 2^−ΔΔCT^ method [41]. Primers used in this study are described at Suppl. Table 2.

### Immunofluorescence

To assess proliferation, immunofluorescence of Ki67 was performed [42]. Cells were fixed with 4% PFA (60 min, RT) and permeabilized with 0.25% Triton-100 (3 min, RT). Nonspecific binding was blocked with 3% BSA (30 min, RT). Cells were incubated with anti-Ki67 antibody or rabbit IgG, polyclonal (isotype control) (overnight, 4 °C) followed by anti-rabbit IgG conjugated with alexa-fluor 594 (1 h, RT). Cells were washed, mounted with SlowFade™ Gold Antifade Mountant with DAPI and observed by fluorescence microscope with images recorded using the Axion Vision program. At least 400 cells were observed per experimental group.

### Apoptosis assay

Apoptosis was assessed by the TUNEL assay, using the In Situ Cell Death Detection Kit according to manufacturer’s protocol [34]. Stained cells were observed by fluorescence microcopy and images were recorded using the Axion Vision program. At least 400 cells were observed per experimental group [21].

### Human samples

Follicular fluid was obtained from single leading follicles of IVF patients at CReATe Fertility Centre, Toronto, Canada, and Taipei Medical University Hospital, Taipei, Taiwan. Serum was obtained from the same patients on the day of oocyte retrieval at Taipei Medical University Hospital. Informed consent was obtained from subjects participated in the present investigation; Veritas IRB (Protocol # 16,518), Taipei Medical University Hospital University Hospital REB (#:TMU-JIRB 201410033) and the Ottawa Hospital REB (Protocol #20,170,453-01H)].

PCOS patients were diagnosed according to Rotterdam criteria [3]. Body mass index (BMI, kg/m^2^) was calculated as weight (kg) divided by height (m) squared. All patients were under 40 years old and normal weight (BMI; 18.5 – 24.9) [43] and had normal thyroid function and prolactin levels, without oligomenorrhea other than PCOS (e.g. hypothyroidism, Cushing’s disease, late onset congenital adrenal hyperplasia). These patients had not received insulin sensitizers (e.g. metformin) for at least three months before IVF treatment started. Since serum NPY levels in obese women are lower than those in non-obese women [44], follicular fluids and serum were collected only from women with normal weight to avoid the confounding influence of obesity. Follicular fluid was obtained from 42 non-PCOS and 23 PCOS patients and serum was from 23 non-PCOS and 10 PCOS patients. Both non-PCOS and PCOS patients were age-matched [non-PCOS: 33 (28-39) vs. PCOS: 32 (21-39), median (range), *p* > 0.05] and BMI-matched (non-PCOS: 22.4 (18.8–24.9) vs. PCOS: 21.0 (18.6–24.9), median (range), *p* > 0.05).

### Measurement of NPY in follicular fluid

Human follicular fluid (FF) and serum were requested and retrieved from the CReATe Fertility biobank and Taipei Medical University Hospital and thawed on ice. FF (100ul) was cleared by centrifugation to remove any precipitate or cell debris (15 min 3,000xg, 4C), and FF and serum were assayed in duplicate for NPY using NPY ELISA kit, according to the manufacturer’s protocol (EMD Millipore Corporation). Absorbance was read at 450 and 650 nm with SpectraMax® 384 Plus microplate reader (Molecular devices, San Jose, CA) and concentrations were calculated using linear regression. The sensitivity of the assay was 3.9 pg/ml, intra and inter assay variabilities were 2.07 ± 0.62% and 0.92 ± 0.78% (mean ± SD), respectively.

### Statistical analysis

Results are analyzed by paired* t* test, unpaired *t* test, Welch’s test, two-way ANOVA with Bonferroni’s post hoc analysis for multiple comparison, or Pearson’s *r* test for correlation test. For comparisons of unpaired samples, the* t* test was used for equal variances by *F* test and the Welch’s test for unequal variances. Statistical analyses were performed using Prism 7 (GraphPad software Inc.). Significant differences were considered at *P* < 0.05.

### Supplementary Information

**Additional file 1.**

## Data Availability

All data is contained in the manuscript.

## Abbreviations

BSA: Bovine serum albumin
eCG: Equine chorionic gonadotropin
FBS: Fetal bovine serum
IVF: In vitro fertilization
NPY: Neuropepetide Y
NPY1R: NPY receptor Y1
NPY2R: NPY receptor Y2
NPY4R: NPY receptor Y4
NPY5R: NPY receptor Y5
PCOS: Polycystic ovarian syndrome
PCR: Polymerase chain reaction
PFA: Paraformaldehyde
RT: Room temperature
