# Supplementary material for: Neuropeptide Y directly reduced apoptosis of granulosa cells, and the expression of NPY and its receptors in PCOS subjects
Source: J Ovarian Res. 2023 Aug 31;16:182. doi: 10.1186/s13048-023-01261-8 (PMC10469470; doi:10.1186/s13048-023-01261-8)
Supplement: Supplementary file 1 — Additional file 1. [file 13048_2023_1261_MOESM1_ESM.docx]

**Supplemental table 1: Reagents and materials used in the present studies**

| Product name | Company |
| --- | --- |
| Folligon (eCG, equine chorionic gonadotropin) | Merck |
| RNeasy minikit | QIAGEN |
| PCR primers | QIAGEN |
| SYBER Green I Master | Roche Diagnostics GmbH |
| cOmplete (a proteinase inhibitor) | Roche Diagnostics GmbH |
| phosSTOP (a phosphatase inhibitor cocktail) | Roche Diagnostics GmbH |
| In Situ Cell Death Detection Kit | Roche Diagnostics GmbH |
| Bovine serum albumin (BSA) | Millipore Sigma |
| Paraformaldehyde (PFA) | Millipore Sigma |
| Phosphate-buffered saline containing 0.05% tween-20 (PBS-T) | Millipore Sigma |
| Ethylene glycol-bis (β-aminoethyl ether)-N,N,N′,N′-tetraacetic acid (EGTA) | Millipore Sigma |
| Sucrose | Millipore Sigma |
| Six-well plate | Corning |
| Eight-chamber slide | Corning |
| High-Capacity cDNA Reverse Transcription Kits | Thermo Fisher Scientific |
| M199 | Thermo Fisher Scientific |
| Penicillin and streptomycin | Thermo Fisher Scientific |
| Amphotericin B | Thermo Fisher Scientific |
| Fetal bovine serum (FBS) | Thermo Fisher Scientific |
| Sodium citrate | Thermo Fisher Scientific |
| SlowFade™ Gold Antifade Mountant with DAPI | Thermo Fisher Scientific |
| Cell lysis buffer | Cell signaling technology |
| Bio-Rad DC Protein Assay Reagent | Bio-Rad Laboratories |
| NPY ELISA kit | EMD Millipore Corporation |
| 5α-dihydrotestosterone | Steraloids |
| Rat neuropeptide Y | Abcam |

**Supplemental table 2: Antibodies used in the present studies**

| Product name | Company | Cat number | Dilution | |
| --- | --- | --- | --- | --- |
|  |  |  | WB | ICC |
| Neuropeptide Y (D7Y5A) XP® Rabbit mAb | Cell Signaling | 11976 | 1:1000 |  |
| Anti-Ki67 antibody | Abcam | ab15580 |  | 1:100 |
| Anti-NPY1R antibody | Abcam | ab216966 | 1:500 |  |
| Anti-Neuropeptide Y2 Receptor | Alomone labs | ANR-022 | 1:500 |  |
| Anti-Neuropeptide Y4 Receptor | Alomone labs | ANR-024 | 1:500 |  |
| Anti-NPY5R antibody | Abcam | ab133757 | 1:15000 |  |
| Anti-GAPDH antibody | Abcam | ab181602 | 1:3000 |  |
| Rabbit IgG, Polyclonal-Isotype Control | Abcam | ab171870 |  |  |
| Goat Anti-Rabbit IgG (H+L)-HRP Conjugate | Bio-Rad | 170-6515 | 1:2000 |  |
| Goat anti-Rabbit IgG (H+L) Cross-Adsorbed Secondary Antibody, Alexa Fluor 594 | Thermo Fisher Scientific | A-11012 |  | 1:200 |

WB: Western blot;

ICC: Immunocytochemistry;

**Supplemental table 2: Primers used in the present studies**

| Target gene | Gene code | Product name | Company | Cat# | Product length (bp) | exon |
| --- | --- | --- | --- | --- | --- | --- |
| *Gapdh* (rat) | NM_017008 (1306 bp) | Rn_Gapd_1_SG | Qiagen | QT00199633 | 149 | 1/3 |
| *BetaActin* (rat) | NM_031144 (1293 bp) | Rn_ActB_1_SG | Qiagen | QT00193473 | 125 | 2/3 |
| *Neuropeptide Y* (rat) | [NM_012614 (567 bp)](http://www.ncbi.nlm.nih.gov/entrez/query.fcgi?holding=&db=Nucleotide&cmd=search&term=NM_012614) | Rn_Npy_1_SG | Qiagen | QT00180355 | 150 | 2/3 |
